# Relationship between air pollution exposure and the progression of idiopathic pulmonary fibrosis in Madrid: Chronic respiratory failure, hospitalizations, and mortality. A retrospective study

**DOI:** 10.3389/fpubh.2023.1135162

**Published:** 2023-03-10

**Authors:** Pablo Mariscal-Aguilar, Luis Gómez-Carrera, Carlos Carpio, Ester Zamarrón, Gema Bonilla, María Fernández-Velilla, Isabel Torres, Isabel Esteban, Rita Regojo, Mariana Díaz-Almirón, Francisco Gayá, Elena Villamañán, Concepción Prados, Rodolfo Álvarez-Sala

**Affiliations:** ^1^Department of Respiratory Medicine, Hospital Universitario La Paz, Madrid, Spain; ^2^Research Institute of Hospital Universitario La Paz (IdiPAZ), Madrid, Spain; ^3^Department of Medicine, Universidad Autónoma de Madrid, Madrid, Spain; ^4^Centro de Investigación Biomédica en Red de Enfermedades Respiratorias, Madrid, Spain; ^5^Department of Rheumatology, Hospital Universitario La Paz, Madrid, Spain; ^6^Department of Radiology, Hospital Universitario La Paz, Madrid, Spain; ^7^Department of Pathological Anatomy, Hospital Universitario La Paz, Madrid, Spain; ^8^Department of Pharmacy, Hospital Universitario La Paz, Madrid, Spain

**Keywords:** air pollution, idiopathic pulmonary fibrosis, chronic respiratory failure, hospital admissions, mortality

## Abstract

**Introduction:**

Air pollution has a significant impact on the morbidity and mortality of various respiratory diseases. However, this has not been widely studied in diffuse interstitial lung diseases, specifically in idiopathic pulmonary fibrosis.

**Objective:**

In this study we aimed to assess the relationship between four major air pollutants individually [carbon monoxide (CO), nitrogen dioxide (NO_2_), ozone (O_3_), and nitrogen oxides (NO_x_)] and the development of chronic respiratory failure, hospitalization due to respiratory causes and mortality in patients with idiopathic pulmonary fibrosis.

**Methods:**

We conducted an exploratory retrospective panel study from 2011 to 2020 in 69 patients with idiopathic pulmonary fibrosis from the pulmonary medicine department of a tertiary hospital. Based on their geocoded residential address, levels of each pollutant were estimated 1, 3, 6, 12, and 36 months prior to each event (chronic respiratory failure, hospital admission and mortality). Data was collected from the air quality monitoring stations of the Community of Madrid located <3.5 km (2.2 miles) from each patient's home.

**Results:**

The increase in average values of CO [OR 1.62 (1.11–2.36) and OR 1.84 (1.1–3.06)], NO_2_ [OR 1.64 (1.01–2.66)], and NO_x_ [OR 1.11 (1–1.23) and OR 1.19 (1.03–1.38)] were significantly associated with the probability of developing chronic respiratory failure in different periods. In addition, the averages of NO_2_, O_3_, and NO_x_ were significantly associated with the probability of hospital admissions due to respiratory causes and mortality in these patients.

**Conclusion:**

Air pollution is associated with an increase in the probability of developing chronic respiratory failure, hospitalization due to respiratory causes and mortality in patients with idiopathic pulmonary fibrosis.

## Introduction

Idiopathic pulmonary fibrosis (IPF) is the most common progressive fibrosing lung disease among idiopathic interstitial pneumonias. IPF is the interstitial lung disease with the worst prognosis, with an approximate median survival from the time of diagnosis of between 3 and 5 years (1–4). There is various prognosis factor of this disease (older age, smoking status, lower lung function…) and some studies recently suggested an association between air pollution and clinical course or mortality of IPF (5–11).

There are numerous short- and long-term effects that air pollution can have on the incidence and prognosis of different respiratory pathologies. The impact of urban air pollution has been associated with alterations in lung maturation and development, increased incidence of chronic obstructive pulmonary disease (COPD), COPD exacerbations, poorly controlled asthma, and increased mortality from respiratory causes (12, 13).

Increased emissions from transportation and industry, the rapid urbanization of different regions and the consumption of energy have currently exposed the respiratory system to an exponential increase in atmospheric pollutants (14). Carbon monoxide (CO), nitrogen dioxide (NO_2_), ozone (O_3_), and nitrogen oxides (NO_x_) are among the most important pollutants to assess in terms of their effect on the respiratory system (15).

Polluted air, when it comes into direct contact with and damages the epithelium of the bronchial tree, can induce epigenetic changes leading to increased collagen deposition and abnormal repair of the affected cells (16, 17). We have found several articles that assess the effects of pollution on IPF. In fact, there are some protocols that have related air pollution with acute exacerbations, lung function and mortality of IPF (5–11).

We hypothesized that major urban air pollutants (CO, NO_2_, O_3_, and NO_x_) could impact individually to the development of chronic respiratory failure and hospitalization due to respiratory causes in patients with IPF and we have not found studies that evaluated it. Furthermore, we also investigated the relationship between air pollution and mortality in patients with IPF with different periods of exposure.

In addition, the length of time studied for high concentrations of CO, NO_2_, O_3_, and NO_x_ to cause these outcomes is heterogeneous in studies and it has not been defined. We aimed to focus on pollution levels over 1, 3, 6, 12, and 36 months before an event because it has been demonstrated that measuring mean air pollution over longer periods often results in stronger associations with lung changes (18–23).

## Materials and methods

### Subjects

Patients included were diagnosed according to the consensus criteria of the American Thoracic Society/European Respiratory Society/Japanese Respiratory Society/Latin American Thoracic Association (1, 2). Patients whose basic data (address, age, sex, complementary tests, and treatments) were not available in the medical records, who failed to attend more than two consecutive visits, or lived more than 3.5 km from the nearest air quality station were excluded. Patients whose home address changed during the study period and those who presented incomplete data during follow-up were withdrawn.

### Protocol

This is a exploratory retrospective panel study of a cohort of patients in follow-up for diffuse interstitial lung disease at the Pulmonary Fibrosis Specialty Clinic of the Hospital Universitario La Paz Pneumology Department. The follow-up period was from 2011 to 2020. Patients were evaluated every 3–4 months in the clinic in compliance with the IPF follow-up protocol established in clinical practice guidelines, collecting different clinical data: a questionnaire to assess dyspnea level, symptoms, diagnostic tests, treatments received, results of complementary tests and diagnoses of other diseases. Patients were considered to be under treatment with antifibrotics when such therapy extended beyond 45 days (24).

### Exposure assessment

The Integral Air Quality System of *Ayuntamiento de Madrid* and *Comunidad de Madrid* provides hourly reports through air quality control stations on the concentrations of NO_2_, O_3_, and NO_x_ measured in μg/m^3^ and CO measured in mg/m^3^ (25, 26). Each patient was assigned air pollutant levels obtained from the surveillance station closest to their home; in the event that the station did not measure a specific pollutant, they were associated with another nearby station provided that this was <3.5 km from their home. The distance between surveillance stations and each patient's residence was determined through the coordinates provided by Google Maps. Distance-related averages were used to relate them. We could not control meteorogical factors (temperature and relative humidity) because we have only found meteorogical data in some stations from *Comunidad de Madrid* (26), while we have not obtain it from the *Ayuntamiento de Madrid* (25).

### Outcome measures

The onset of chronic respiratory failure was defined as those patients who had a partial pressure of oxygen of <60 mmHg in baseline arterial blood gases (27). Hospitalizations due to respiratory causes were specified based on the diagnosis reflected in patients' medical record at the time of each admission. In addition, every patient who had a hospitalization were made a respiratory virus test (Influenza, Respiratory Syncytial Virus or SARS-CoV2) and before each hospital admission. Data related to mortality were obtained from each patient's medical record.

Baseline arterial blood gases were obtained by puncture of the radial artery, after ensuring the existence of adequate collateral circulation using the Allen maneuver, in accordance with SEPAR (Spanish Society of Pneumology and Thoracic Surgery) recommendations (28). The arterial blood sample obtained while the patients were breathing room air was measured with an ABL90 blood gas analyzer (Radiometer Medical ApS, Brønshøj, Denmark).

### Ethics

This study was conducted in accordance with the standards of good clinical practice and the ethical principles of the Declaration of Helsinki. It was approved by the La Paz University Hospital Clinical Research Ethics Committee (Code PI-3742, June 20, 2019). Given the retrospective nature of the study, informed consent was not required.

### Statistical method

To calculate the value of each pollutant relative to the period between patient visits, the average of each pollutant was taken, weighted by the inverse of the squared of the distance from the patient's home to the nearest air quality station (maximum distance of 3.5 km).

Correlated data were analyzed using a generalized linear mixed model with the restricted maximum pseudo-likelihood method. Three events were assessed: chronic respiratory failure, hospitalization due to respiratory causes and mortality. A random intercept and unstructured covariance matrix were added to the generalized linear mixed model with binomial distribution and logit link function to estimate the “likelihood of the event”. Each air pollutant was added to the model, estimating the relationship with the binary outcome in terms of the OR. To estimate the average values of air pollutants, a random intercept and unstructured covariance matrix were added to the generalized linear mixed model with normal distribution and an identity link function.

A Poisson log-linear model with robust standard errors (“sandwich” method) was used to study the association between air pollutants and the risk ratio of two events: hospitalization due to respiratory causes and mortality. Non-linear associations for each air pollutant with a risk ratio were tested using a likelihood ratio test comparing a model with the exposure fitted on a spline with a model assuming a linear exposure-outcome relationship. Spline smoothness was selected after determining two and three degrees of freedom. The *p*-value for non-linearity of <0.05 suggests evidence against the linearity assumption.

Three multivariate analysis were calculated (once for each outcome, chronic respiratory failure, hospitalizations and mortality). The Generalized Linear Mixed Model (GLMM) with the Restricted Maximum Pseudo-Likelihood Method (RMPL) was considered to estimate “Probability of the event” with a binomial distribution and logit link function.

Seasons have been controlled and no significant differences were found between hospitalizations and mortality in each period. We have taken two definitions of each season. The first one based on Sesé et al. (8), who defined “cool season” from November to April. Secondly, it has been taken the definition according to Johannson et al. (6) and Dales et al. (29) who stablished “cool season” from October to March. In both cases there were no significant differences between hospitalizations and mortality (Supplementary Tables 1–4). In addition, in order to include season as a potential confounder, seasons have been incorporated in multivariate analyses as categorical regressor variable in the Generalized Linear Mixed Model where it has been taken “cool season” definition from October to March and “warm season” from April to September (6, 29).

On the other hand, statistical models used were also adjusted for age, sex, smoking status, FVC, and DLCO at the time of the first visit, antifibrotic treatment regimen and pulmonary hypertension.

In general, two-tailed *p*-values of <0.05 were considered statistically significant. Analyses were conducted in R Statistical Software V4.0.4 (2021-02-15) with the mgcv package (mgcv_1. 8-33) and SAS Enterprise Guide 8.2 statistical program (SAS Institute Inc., Cary, North Carolina).

## Results

Of the 71 patients initially included in the cohort, two whose place of residence was unknown and one who lived more than 3.5 km from the station were excluded. The baseline characteristics of all included patients may be observed in Table 1. Of the 69 enrolled patients with IPF, 22 developed chronic respiratory failure, 29 were hospitalized at least once for respiratory causes, and 22 died. Results were obtained with calculations adjusted for age, sex, smoking status, lung function and antifibrotic treatment. Of the 22 patients who developed chronic respiratory failure, 13 (59%) were hospitalized due to respiratory causes at least once, and 16 died (72.7%). Of the 29 patients who were hospitalized at least once, 13 (44.8%) developed chronic respiratory failure and 15 (51.7%) died. Finally, of 22 patients who died, 15 (68.1%) had at least one hospital admission and 16 (72.7%) developed chronic respiratory failure. here were significant differences between hospitalizations and mortality. On the other hand, there were too significant differences between chronic respiratory failure and mortality. Patient exposure levels appear in Supplementary Table 5.

**Table 1:** Baseline characteristics of IPF patients.

**Baseline characteristics**	**Mean ±standard deviation or number (percentage)**
**Sex, number (%)**	
Women, number (%)	16 (23.2)
Men, number (%)	53 (76.8)
Age, years	73.70 ± 7.72
Height, cm	72.85 ± 14.12
Weight, kg	165.5 ± 10.32
Body mass index, Kg/m^2^	26.40 ± 3.92
**Smokers, number (%)**	
Non-smoker	39 (56.5)
Current smoker	30 (43.5)
Packs per year	22.65 ±13.97
Charlson Index	2 ± 1.5
Dyspnea (modified Medical Research Council)	1 ± 0.65
Total lung capacity (TLC), % predicted	77.69 ± 14.76
Pulmonary carbon monoxide diffusing capacity (DLCO), % predicted	63.45 ± 20.54
Carbon monoxide transfer coefficient (KCO), % predicted	91.8 ± 17.9
Forced expiratory volume in the first second (FEV_1_) % prebronchodilator, % predicted	88.20 ± 18.3
Forced vital capacity (FVC) % prebronchodilator, % predicted	82.42 ± 18.39
6 min walk test, m	488.89 ± 101.69
Initial oxygen saturation, %	94 ± 2.2
Final oxygen saturation, %	85.83 ± 5.2
pH	7.42 ± 0.01
Partial pressure of oxygen in baseline arterial blood, mmHg	72.40 ± 10
Partial pressure of carbon dioxide in baseline arterial blood, mmHg	37.4 ± 4.1
Systolic pulmonary artery pressure (sPAP)	32.6 ± 7.16
Antifibrotic treatment > 45 days	61 (88.4)
Pirfenidone > 45 days	48 (69.5)
Nintedanib > 45 days	21 (30.5)
Chronic respiratory failure	22 (31.9)
Hospitalizations (respiratory cause)	29 (42)
Deaths	22 (31.9)

### Effects of pollution on the development of chronic respiratory failure

The increment in the average values of CO, NO_2_, and NO_x_ was significantly associated with an increase in the probability of the development of chronic respiratory failure in different periods. In the case of CO, an OR of 1.62 (1.11–2.36) was observed for each 0.1 mg/m^3^ (*p* = 0.01) increase during the 3 months prior to the event and an OR of 1.84 (1.1–3.06) over the previous 6 months (Supplementary Figure 1). Regarding NO_2_, an OR of 1.65 (1.01–2.66) (*p* = 0.04) was obtained for each 10 μg/m^3^ increase with exposure 6 months before the event (Supplementary Figure 2). NO_x_ yielded results with an OR of 1.12 (1.01–1.23) (*p* = 0.03), and OR 1.20 (1.03–1.38) (*p* = 0.01) for each 10 μg/m^3^ increase at 3 and 6 months of exposure prior to the event, respectively (Supplementary Figure 3). Regarding multivariate analysis, the association between each pollutant and chronic respiratory failure was not affected by the inclusion of other pollutants in the models. In addition, there was no effect of seasons in these results (Supplementary Tables 6–10).

### Effects of pollution on admissions due to respiratory causes

The increase in the averages of NO_2_, O_3_, and NO_x_ were significantly associated with an increase in the probability of hospital admission due to respiratory causes in these patients. In the case of NO_2_, a significant relationship was obtained with an exposure of 6, 12, and 36 months before admission (Supplementary Figures 4–6). The positive results obtained with respect to O_3_ involved an exposure of 1 month prior to admission (Supplementary Figure 7). In the case of NO_x_ the association between the probability of hospitalization for respiratory causes occurred with an exposure of 6, 12, and 36 months (Supplementary Figures 8–10). Regarding multivariate analysis, the association between each pollutant and hospitalizations due to respiratory causes was not affected by the inclusion of other pollutants in the models. In addition, there was no effect of seasons in these results (Supplementary Tables 11–15).

### Effects of pollution on mortality

Increases in the averages of NO_2_, O_3_, and NO_x_ were significantly associated with an increase in the probability of death in patients with an exposure to these pollutants of 1 and 3 months in the case of O_3_ and of 12 and 36 months in the case of NO_2_ and NO_x_ (Supplementary Figures 11–16). Regarding multivariate analysis, the association between each pollutant and mortality was not affected by the inclusion of other pollutants in the models. In addition, there was no effect of seasons in these results (Supplementary Tables 16–20).

## Discussion

Our data show that air pollution may be associated with the development of chronic respiratory failure irrespective of other factors such as sex, age, smoking status, lung function, or antifibrotic therapy in patients with IPF. The results obtained indicate that increases in the average values of CO, NO_2_ and NO_x_ are related to an increased probability of the development of chronic respiratory failure in different periods of exposure (three and 6 months prior to the event).

To date, we have found no works in the literature consulted that study the association between the pollution caused by CO, NO_2_, and NO_x_ and the probability of developing chronic respiratory failure in patients with IPF. However, we have identified protocols that do so with variations in oxygen saturation whose conclusions partially coincide with those of our study (30, 31). Such is the case of work carried out by DeMeo et al. (30) who demonstrated that for each 11.45 μg/m^3^ increase in PM_2.5_ oxygen saturation decreased by 0.172% (−0.313 to −0.031). Similar research developed by Luttmann-Gibson et al. (31) analyzing NO_2_, SO_2_, PM_2.5_, O_3_, SO_4_^2−^, and elemental carbon concluded that an increase of 13.4 μg/m^3^ in PM_2.5_ on the previous day was associated with a decrease of −0.18% (−0.31 to −0.06) in oxygen saturation, while an increase of 5.1 μg/m^3^ in SO_4_^2−^ was associated with a decrease of −16% (−0.27 to −0.04) in pulse oximetry.

The possible disparity in results may be due to all protocols we consulted examined oxygen saturation measured by pulse oximetry, while we used baseline arterial blood gases, which is more accurate (28). Secondly, our study included a larger sample size compared to the subjects included in these studies. In addition, our study used stations close to patients' homes, while DeMeo et al. (30) only monitored pollution data at the study site.

Regarding to hospitalizations due to respiratory deterioration we observed that increases in NO_2_, O_3_, and NO_x_ were significantly associated with the increase in the number of hospitalizations for this cause in patients with IPF with relatively long periods of exposure compared to other studies (5, 6, 8, 29). We have found no research that examines the association between pollution and the probability of hospitalization due to respiratory causes in patients with IPF. However, we have observed that there are studies (5, 6, 8, 29) that analyze other variables.

Our results regarding NO_2_ and O_3_ partially coincide with those obtained by Dales et al. (29) who obtained a significant association between the levels of CO_2_, NO_2_, SO_2_, PM_2.5_, PM_10_, and O_3_ and hospitalizations although they do not do so in the same time interval. This may be due to the fact that this study measured not only admissions due to respiratory causes, as in our work, but hospitalizations of patients with IPF due to any pathology, which may constitute a confounding factor with respect to the exposure time necessary for the disease to lead to hospital admission. Tomos et al. (5) obtained similar results to our study because of the impact of NO_2_ in the increase of exacerbations. On the other hand, the results obtained in our protocol correspond more closely with those found by Johannson et al. (6) who demonstrated that pollution caused by increased levels of NO_2_ and O_3_ increased the risk of acute exacerbations. Finally, Sesé et al. (8) found consistent results relating to O_3_ levels and acute exacerbations. Nevertheless, although they did not examined the same exposure period and they have only assessed exacerbations of IPF. The differences in outcomes could be due to dissimilarities in the data sources, as well as in the composition of the air, which may have influenced the time course of these particles leading to patient hospitalization for IPF.

The increase in hospitalizations due to pollutants that we have detected may be supported by the effect that these pollutants have on the respiratory system. NO_2_ and NO_x_ derive from the combustion of motorized vehicles and can reach the lower airway, causing inflammation and changes in its caliber. Finally, excessive ozone can decrease ventilatory capacity (32). These demonstrated effects of pollutants in the respiratory system add more power to our data, since they are highly consistent.

Our data indicate that the action of certain pollutants, namely NO_2_, O_3_, and NO_x_ may have a significant impact on the mortality of these patients with different exposure times. In this regard, we have found several works in the literature that evaluate IPF mortality and pollution with some discrepancies in results. Sesé et al. (8) did not find positive results in NO_2_, and O_3_ maybe because they had different sample size characteristics with more mortality than ours (53.4 vs. 31.9%) and, as same as the study of Johannson et al. (6), they used 42-day exposure time, which could be insufficient time for the pollutants to contribute to increasing the mortality of patients with IPF. In addition, Mariscal et al. (33) reported an increased risk of mortality with CO and this results differ with our protocol because the measurement was from a single surveillance station in central Madrid while in ours we have used all the stations in the region. Finally, Yoon et al. (11), with a larger sample size, observed a clear influence of NO_2_ levels on the mortality of these patients.

Discrepancies may also be due to the different levels of exposure and toxicity, the study populations in different climatic zones, and the criteria for diagnosing and admitting patients with IPF. Therefore, although these works have similar objectives, the comparison of results must be performed with caution, since the methodology was different.

Regarding the correspondence between our study outcomes and those of Yoon et al. (11), we believe that our protocol coincides in the finding that NO_2_ is the pollutant most associated with the deterioration of IPF, whether in terms of chronic respiratory failure, hospital admissions or mortality. Therefore, it seems reasonable to think that this particle, which is linked to road traffic, causes inflammation and an increase in surface tension in the alveoli that contributes to the progression of IPF in various aspects. This suggests that a correct action in reducing the levels of NO_2_, O_3_, and NO_x_ through stricter control of traffic and motor vehicle emissions could promote a better clinical course for IPF patients residing in cities with high pollution levels.

This research project has several limitations. First, it is a single-center study with a limited number of subjects, insofar as it is a low prevalence disease, although the cohort comes from an interstitial lung disease unit with a not insignificant sample size. Second, exposure to air pollution in the workplace, which could play an important role in this type of study, was not taken into account. No information on temperature and relative humidity was available. Regarding potential confounding factors, it was not possible to control for all of them, since there are many more particles in the air than those studied in this protocol, which could have prevented the demonstration of other effects of air pollution. Finally, although 3.5 km was the best available exposure assessment in our protocol, an important limitation of the study was the error in the exposure measurements.

However, our study also has its strengths. It is a work that simultaneously analyzes a greater number of pollutants than most other published protocols (5–11) in this case up to four (CO, NO_2_, O_3_, and NO_x_), selected according to the evidence available in this type of research.

In addition, the indicators assessed (blood gas, hospitalizations, and mortality) are decisive in the clinical course of patients with idiopathic pulmonary fibrosis and to the best of our knowledge there is no other article that considers all these variables together. Our work also includes the averages of each pollutant during 1, 3, 6, 12, and 36 months so this results could be useful to hypothesize with caution that both short and long term exposure to air pollution could be harmful to the clinical course of the patient (18–22).

The foregoing adds significant value to this protocol analyzing the impact of environmental pollution on the clinical course and mortality of idiopathic pulmonary fibrosis.

## Conclusions

In conclusion, the findings of this study suggest that environmental pollution, specifically that caused by four main pollutants (CO, NO_2_, O_3_, and NO_x_), can increase cases of chronic respiratory failure, hospitalizations due to respiratory causes and mortality in patients with IPF. These results are in line with the WHO recommendations for reducing polluting emissions and could encourage changes in environmental policies to improve the follow-up and clinical course of these patients. It is necessary to continue this line of research with future protocols that allow for more precise quantifications of ambient air quality, paying special attention to these pollutants.

## Data availability statement

The original contributions presented in the study are included in the article/Supplementary material, further inquiries can be directed to the corresponding author.

## Ethics statement

The studies involving human participants were reviewed and approved by the La Paz University Hospital Clinical Research Ethics Committee (Code PI-3742, June 20, 2019). Given the retrospective nature of the study, informed consent was not required. Written informed consent for participation was not required for this study in accordance with the national legislation and the institutional requirements.

## Author contributions

Conceptualization: PM-A, LG-C, CP, and RÁ-S. Methodology: PM-A, CC, LG-C, MD-A, FG, and RÁ-S. Software and formal analysis: PM-A, CC, MD-A, and FG. Validation: CC, IT, PM-A, MF-V, and RÁ-S. Research: LG-C, CC, EZ, EV, and RÁ-S. Resources: CC, EZ, PM-A, MD-A, FG, and RÁ-S. Data curation: PM-A and GB. Writing—original draft preparation: PM-A and RÁ-S. Writing—review and editing: CC, PM-A, IE, RR, and RÁ-S. Visualization: CC, PM-A, CP, and RÁ-S. Supervision: LG-C, CC, and RÁ-S. All authors contributed to the article and approved the submitted version.
