# Supplementary material for: Relationship between air pollution exposure and the progression of idiopathic pulmonary fibrosis in Madrid: Chronic respiratory failure, hospitalizations, and mortality. A retrospective study
Source: Front Public Health. 2023 Mar 10;11:1135162. doi: 10.3389/fpubh.2023.1135162 (PMC10036896; doi:10.3389/fpubh.2023.1135162)
Supplement: Supplementary file 1 [file Data_Sheet_1.pdf]

Supplementary Tables

Relationship between air pollution exposure and the progression of idiopathic pulmonary fibrosis in Madrid: Chronic respiratory failure, hospitalizations and mortality. A retrospective study

Pablo Mariscal-Aguilar1,2,3,4*
Luis Gómez-Carrera1,2,3,4
Carlos Carpio1,2,3,4
Ester Zamarrón1,2,3,4
Gema Bonilla2,3,5
María Fernández-Velilla2,3,6
Isabel Torres2,3,6
Isabel Esteban2,3,7
Rita Regojo2,3,7
Mariana Díaz-Almirón2
Francisco Gayá2
Elena Villamañán2,3,8
Concepción Prados1,2,3,4
Rodolfo Álvarez-Sala 1,2,3,4

1. Department of Respiratory Medicine, Hospital Universitario La Paz, Madrid, Spain.

2. Research Institute of Hospital Universitario La Paz (IdiPAZ), Madrid, Spain.

3. Universidad Autónoma de Madrid, Madrid, Spain.

4. Centro de Investigación Biomédica en Red de Enfermedades Respiratorias (CIBERES).

5. Department of Rheumatology, Hospital Universitario La Paz, Madrid, Spain.

6. Department of Radiology, Hospital Universitario La Paz, Madrid, Spain.

7. Department of Pathological Anatomy, Hospital Universitario La Paz, Madrid, Spain.

8. Department of Pharmacy, Hospital Universitario La Paz, Madrid, Spain.

*Corresponding author: Pablo Mariscal Aguilar
Email: pmaguilar91@gmail.com

Keywords: Air pollution, idiopathic pulmonary fibrosis, chronic respiratory failure, hospital admissions, mortality.

Hospitalizations			
	**NO**	**YES**	***Marginal Row Totals***
**Cool season, November to April**	26 (59%)	18 (41%)	44
**Warm season, May to October**	14 (56%)	11 (44%)	25
Marginal Column Totals	40	29	69 (Grand Total)

The chi-square statistic is 0.0625. The p-value is .80257. Not significant at $p < .05$.
The chi-square statistic with Yates correction is 0. The p-value is .997066. Not significant at $p < .05$.

Supplementary table 1. Seasonal cofounding factor adjustment
No table of figures entries found.

Mortality			
	**NO**	**YES**	***Marginal Row Totals***
**Cool season, November to April**	29 (76.3%)	9 (23.7%)	38 (100%)
**Warm season, May to October**	18 (58.1%)	13 (41.9%)	31 (100%)
Marginal Column Totals	47	22	69 (Grand Total)

The chi-square statistic is 2.6185. The p-value is .105621. Not significant at $p < .05$.
The chi-square statistic with Yates correction is 1.8456. The p-value is .174296. Not significant at $p < .05$.

Supplementary table 2. Seasonal cofounding factor adjustment

Hospitalizations			
	**NO**	**YES**	***Marginal Row Totals***
**Cool season, October to March**	17 (65.4%)	9 (34.6%)	26 (100%)
**Warm season, April to September**	23 (53.5%)	20 (46.5%)	43 (100%)
Marginal Column Totals	40	29	69 (Grand Total)

The chi-square statistic is 0.9411. The *p*-value is .331986. *Not* significant at $p < .05$.
The chi-square statistic with Yates correction is 0.5162. The *p*-value is .472465. *Not* significant at $p < .05$.

Supplementary table 3. Seasonal cofounding factor adjustment

Mortality			
	**NO**	**YES**	***Marginal Row Totals***
**Cool season, October to March**	28 (75.7%)	9 (24.3%)	37 (100%)
**Warm season, April to September**	19 (59.4%)	13 (40.6%)	32 (100%)
Marginal Column Totals	47	22	69 (Grand Total)

The chi-square statistic is 2.0994. The p-value is .147359. *Not* significant at $p < .05$. The chi-square statistic with Yates correction is 1.4159. The p-value is .234078. *Not* significant at $p < .05$.

Supplementary table 4. Seasonal cofounding factor adjustment

	1 month	3 months	6 months	12 months	36 months
CO (mg/m^3)	0,39 ± 0,17	0,36 ± 0,11	0,36 ± 0,08	0,35 ± 0,07	0,36 ± 0,04
NO_2 ($\mu g/m^3$)	34,95 ± 15,23	32,83 ± 11,25	33,48 ± 8,36	33,47 ± 5,97	36,96 ± 5,16
O_3 ($\mu g/m^3$)	45,91 ± 21,36	48,48 ± 16,87	50,24 ± 11,64	54,20 ± 4,54	53,37 ± 4,10
NO_X ($\mu g/m^3$)	67,06 ± 43,73	60,80 ± 30,91	61,61 ± 23,32	60,01 ± 15,33	69 ± 14,1

Supplementary table 5. Accumulated averages of each pollutant in the first visit (mean ± standard deviation)

Chronic respiratory failure 1 month exposure					
**Effect**	**Estimate**	**Standard error**	**DF**	**t valor**	**Pr > |t|**
**Intercept**	7.0108	3.4254	38	2.05	0.0476
**NO_2**	-0.02499	0.06164	102	-0.41	0.6860
**CO**	0.01408	0.04274	102	0.33	0.7424
**O_3**	-0.05383	0.03369	102	-1.60	0.1132
**NO_x**	-0.00747	0.02061	102	-0.36	0.7177
**Basal DLCO**	-0.07546	0.01988	102	-3.80	0.0003
**Cool seasons**	-0.9274	0.9421	102	-0.98	0.3272

Supplementary table 6. Chronic respiratory failure multivariate analysis (one month exposure)

**Chronic respiratory failure 3 months exposure**					
**Effect**	**Estimate**	**Standard error**	**DF**	**t valor**	**Pr > |t|**
**Intercept**	4.4957	4.0230	38	1.12	0.2708
**NO_2**	-0.08029	0.08601	102	-0.93	0.3527
**CO**	0.001774	0.06061	102	0.03	0.9767
**O_3**	-0.02807	0.03568	102	-0.79	0.4334
**NO_x**	0.03730	0.03103	102	1.20	0.2321
**Basal DLCO**	-0.06451	0.01945	102	-3.32	0.0013
**Cool seasons**	-1.3986	0.7652	102	-1.83	0.0705

Supplementary table 7. Chronic respiratory failure multivariate analysis (3 months exposure)

**Chronic respiratory failure 6 months exposure**					
**Effect**	**Estimate**	**Standard error**	**DF**	**t valor**	**Pr > |t|**
**Intercept**	-0.1578	5.4351	38	-0.03	0.9770
**NO_2**	-0.01460	0.1205	102	-0.12	0.9038
**CO**	-0.01238	0.07521	102	-0.16	0.8696
**O_3**	0.01202	0.04312	102	0.28	0.7810
**NO_x**	0.03156	0.03920	102	0.81	0.4226
**Basal DLCO**	-0.05956	0.01921	102	-3.10	0.0025
**Cool seasons**	0.05716	0.4632	102	0.12	0.9020

Supplementary table 8. Chronic respiratory failure multivariate analysis (6 months exposure)

Chronic respiratory failure 12 months exposure					
**Effect**	**Estimate**	**Standard error**	**DF**	**t valor**	**Pr > |t|**
**Intercept**	-2.9991	9.4167	38	-0.32	0.7519
**NO_2**	-0.01652	0.1640	102	-0.10	0.9200
**CO**	-0.1232	0.1368	102	-0.90	0.3700
**O_3**	0.08056	0.09915	102	0.81	0.4184
**NO_x**	0.07679	0.05875	102	1.31	0.1941
**Basal DLCO**	-0.06274	0.01904	102	-3.30	0.0014
**Cool seasons**	0.1931	0.4510	102	0.43	0.6694

Supplementary table 9. Chronic respiratory failure multivariate analysis (12 months exposure)

Chronic respiratory failure 36 months exposure					
**Effect**	**Estimate**	**Standard error**	**DF**	**t valor**	**Pr > |t|**
**Intercept**	-19.3497	12.8713	38	-1.50	0.1410
**NO_2**	0.4277	0.2674	102	1.60	0.1128
**CO**	-0.3119	0.2687	102	-1.16	0.2485
**O_3**	0.2797	0.1850	102	1.51	0.1335
**NO_x**	0.01478	0.07837	102	0.19	0.8508
**Basal DLCO**	-0.06531	0.01904	102	-3.43	0.0009
**Cool seasons**	0.2532	0.4533	102	0.56	0.5777

Supplementary table 10. Chronic respiratory failure multivariate analysis (36 months exposure)

Hospitalizations 1 month exposure					
**Effect**	**Estimate**	**Standard error**	**DF**	**t valor**	**Pr > |t|**
**Intercept**	6.7646	5.1985	37	1.30	0.2012
**NO_2**	-0.03776	0.1335	37	-0.28	0.7788
**CO**	0.000531	0.08861	37	0.01	0.9953
**O_3**	-0.03802	0.03669	37	-1.04	0.3067
**NO_x**	-0.01252	0.05779	37	-0.22	0.8297
**Basal DLCO**	-0.04261	0.02030	37	-2.10	0.0427
**Cool seasons**	-0.3211	0.7027	37	-0.46	0.6504

Supplementary table 11. Hospitalizations multivariate analysis (one month exposure)

Hospitalizations 3 months exposure					
**Effect**	**Estimate**	**Standard error**	**DF**	**t valor**	**Pr > |t|**
**Intercept**	3.7971	6.6725	37	0.57	0.5728
**NO_2**	0.01159	0.1454	37	0.08	0.9369
**CO**	0.003819	0.08456	37	0.05	0.9642
**O_3**	-0.02119	0.04655	37	-0.46	0.6516
**NO_x**	-0.01206	0.04498	37	-0.27	0.7901
**Basal DLCO**	-0.04029	0.01955	37	-2.06	0.0464
**Cool seasons**	-0.3726	0.7117	37	-0.52	0.6037

Supplementary table 12. Hospitalizations multivariate analysis (3 months exposure)

Hospitalizations 6 months exposure					
**Effect**	**Estimate**	**Standard error**	**DF**	**t valor**	**Pr > |t|**
**Intercept**	-1.8817	7.3151	37	-0.26	0.7984
**NO_2**	0.1450	0.1797	37	0.81	0.4250
**CO**	0.1163	0.1086	37	1.07	0.2912
**O_3**	-0.01067	0.06387	37	-0.17	0.8682
**NO_x**	-0.06381	0.07253	37	-0.88	0.3846
**Basal DLCO**	-0.04375	0.01987	37	-2.20	0.0340
**Cool seasons**	-0.3955	0.7366	37	-0.54	0.5945

Supplementary table 13. Hospitalizations multivariate analysis (6 months exposure)

Hospitalizations 12 months exposure					
**Effect**	**Estimate**	**Standard error**	**DF**	**t valor**	**Pr > |t|**
**Intercept**	-1.2843	8.1065	37	-0.16	0.8750
**NO_2**	0.2182	0.2144	37	1.02	0.3154
**CO**	0.1667	0.1812	37	0.92	0.3637
**O_3**	-0.04490	0.1228	37	-0.37	0.7167
**NO_x**	-0.1123	0.08632	37	-1.30	0.2015
**Basal DLCO**	-0.04285	0.01990	37	-2.15	0.0379
**Cool seasons**	-0.2687	0.7434	37	-0.36	0.7199

Supplementary table 14. Hospitalizations multivariate analysis (12 months exposure)

Hospitalizations 36 months exposure					
**Effect**	**Estimate**	**Standard error**	**DF**	**t valor**	**Pr > |t|**
**Intercept**	-9.2419	12.8179	37	-0.72	0.4754
**NO_2**	0.3253	0.2678	37	1.21	0.2322
**CO**	0.2344	0.2503	37	0.94	0.3551
**O_3**	0.01868	0.1481	37	0.13	0.9003
**NO_x**	-0.1324	0.09084	37	-1.46	0.1533
**Basal DLCO**	-0.05128	0.02250	37	-2.28	0.0285
**Cool seasons**	-0.3413	0.7405	37	-0.46	0.6475

Supplementary table 15. Hospitalizations multivariate analysis (36 months exposure)

**Mortality 1 month exposure**					
**Effect**	**Estimate**	**Standard error**	**DF**	**t valor**	**Pr > |t|**
**Intercept**	-6.5906	7.5064	37	-0.88	0.3856
**NO_2**	0.2688	0.2023	37	1.33	0.1920
**CO**	0.1247	0.1520	37	0.82	0.4173
**O_3**	0.05368	0.04867	37	1.10	0.2772
**NO_x**	-0.08398	0.08436	37	-1.00	0.3260
**Basal DLCO**	-0.1007	0.03476	37	-2.90	0.0063
**Cool seasons**	0.1521	0.8432	37	0.18	0.8579

Supplementary table 16. Mortality multivariate analysis (one month exposure)

Mortality 3 months exposure					
**Effect**	**Estimate**	**Standard error**	**DF**	**t valor**	**Pr > |t|**
**Intercept**	-6.1016	8.4480	37	-0.72	0.4747
**NO_2**	0.1861	0.2021	37	0.92	0.3629
**CO**	0.002657	0.1139	37	0.02	0.9815
**O_3**	0.07123	0.05875	37	1.21	0.2331
**NO_x**	-0.01673	0.06343	37	-0.26	0.7934
**Basal DLCO**	-0.08337	0.02913	37	-2.86	0.0069
**Cool seasons**	0.3068	0.8430	37	0.36	0.7180

Supplementary table 17. Mortality multivariate analysis (3 months exposure)

Mortality 6 months exposure					
**Effect**	**Estimate**	**Standard error**	**DF**	**t valor**	**Pr > |t|**
**Intercept**	-7.4164	10.1076	37	-0.73	0.4677
**NO_2**	0.04676	0.1990	37	0.24	0.8155
**CO**	-0.03341	0.1378	37	-0.24	0.8098
**O_3**	0.1204	0.09347	37	1.29	0.2056
**NO_x**	0.06933	0.07728	37	0.90	0.3754
**Basal DLCO**	-0.1064	0.03554	37	-3.00	0.0049
**Cool seasons**	0.5009	0.8907	37	0.56	0.5772

Supplementary table 18. Mortality multivariate analysis (6 months exposure)

Mortality 12 months exposure					
**Effect**	**Estimate**	**Standard error**	**DF**	**t valor**	**Pr > |t|**
**Intercept**	-7.0519	9.6188	37	-0.73	0.4681
**NO_2**	-0.06014	0.2297	37	-0.26	0.7949
**CO**	-0.3404	0.2539	37	-1.34	0.1882
**O_3**	0.2563	0.1647	37	1.56	0.1282
**NO_x**	0.1747	0.1123	37	1.56	0.1282
**Basal DLCO**	-0.1111	0.03722	37	-2.99	0.0050
**Cool seasons**	0.5120	0.9130	37	0.56	0.5783

Supplementary table 19. Mortality multivariate analysis (12 months exposure)

Mortality 36 months exposure					
**Effect**	**Estimate**	**Standard error**	**DF**	**t valor**	**Pr > |t|**
**Intercept**	-15.6428	18.0378	37	-0.87	0.3914
**NO_2**	0.2028	0.4295	37	0.47	0.6395
**CO**	-0.4114	0.5881	37	-0.70	0.4886
**O_3**	0.3574	0.2716	37	1.32	0.1963
**NO_x**	0.1147	0.1843	37	0.62	0.5377
**Basal DLCO**	-0.1078	0.03479	37	-3.10	0.0037
**Cool seasons**	0.6354	0.9562	37	0.66	0.5105

Supplementary table 20. Mortality multivariate analysis (36 months exposure)
