# Supplementary material for: Relationship between air pollution exposure and the progression of idiopathic pulmonary fibrosis in Madrid: Chronic respiratory failure, hospitalizations, and mortality. A retrospective study
Source: Front Public Health. 2023 Mar 10;11:1135162. doi: 10.3389/fpubh.2023.1135162 (PMC10036896; doi:10.3389/fpubh.2023.1135162)
Supplement: Supplementary file 2 [file Data_Sheet_2.pdf]

Supplementary Figures

Relationship between air pollution exposure and the progression of idiopathic pulmonary fibrosis in Madrid: Chronic respiratory failure, hospitalizations and mortality. A retrospective study

Pablo Mariscal-Aguilar[1,2,3,4*]
Luis Gómez-Carrera[1,2,3,4]
Carlos Carpio[1,2,3,4]
Ester Zamarrón[1,2,3,4]
Gema Bonilla[2,3,5]
María Fernández-Velilla[2,3,6]
Isabel Torres[2,3,6]
Isabel Esteban[2,3,7]
Rita Regojo[2,3,7]
Mariana Díaz-Almirón[2]
Francisco Gayá[2]
Elena Villamañán[2,3,8]
Concepción Prados[1,2,3,4]
Rodolfo Álvarez-Sala [1,2,3,4]

1. Department of Respiratory Medicine, Hospital Universitario La Paz, Madrid, Spain.

2. Research Institute of Hospital Universitario La Paz (IdiPAZ), Madrid, Spain.

3. Universidad Autónoma de Madrid, Madrid, Spain.

4. Centro de Investigación Biomédica en Red de Enfermedades Respiratorias (CIBERES).

5. Department of Rheumatology, Hospital Universitario La Paz, Madrid, Spain.

6. Department of Radiology, Hospital Universitario La Paz, Madrid, Spain.

7. Department of Pathological Anatomy, Hospital Universitario La Paz, Madrid, Spain.

8. Department of Pharmacy, Hospital Universitario La Paz, Madrid, Spain.

*Corresponding author: Pablo Mariscal Aguilar
Email: pmaguilar91@gmail.com

Keywords: Air pollution, idiopathic pulmonary fibrosis, chronic respiratory failure, hospital admissions, mortality.

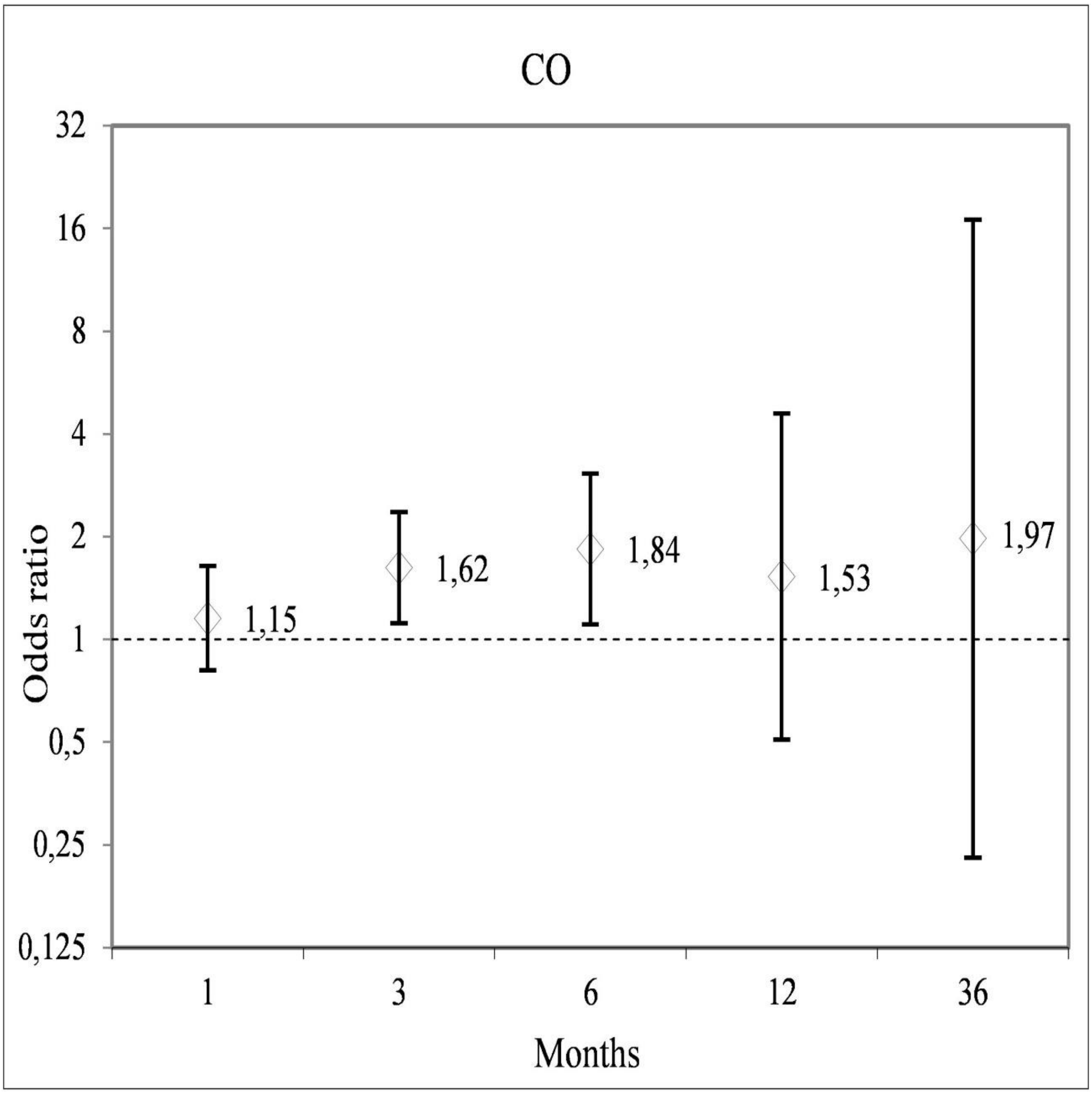

Supplementary figure 1. Effect of CO on the development of chronic respiratory failure

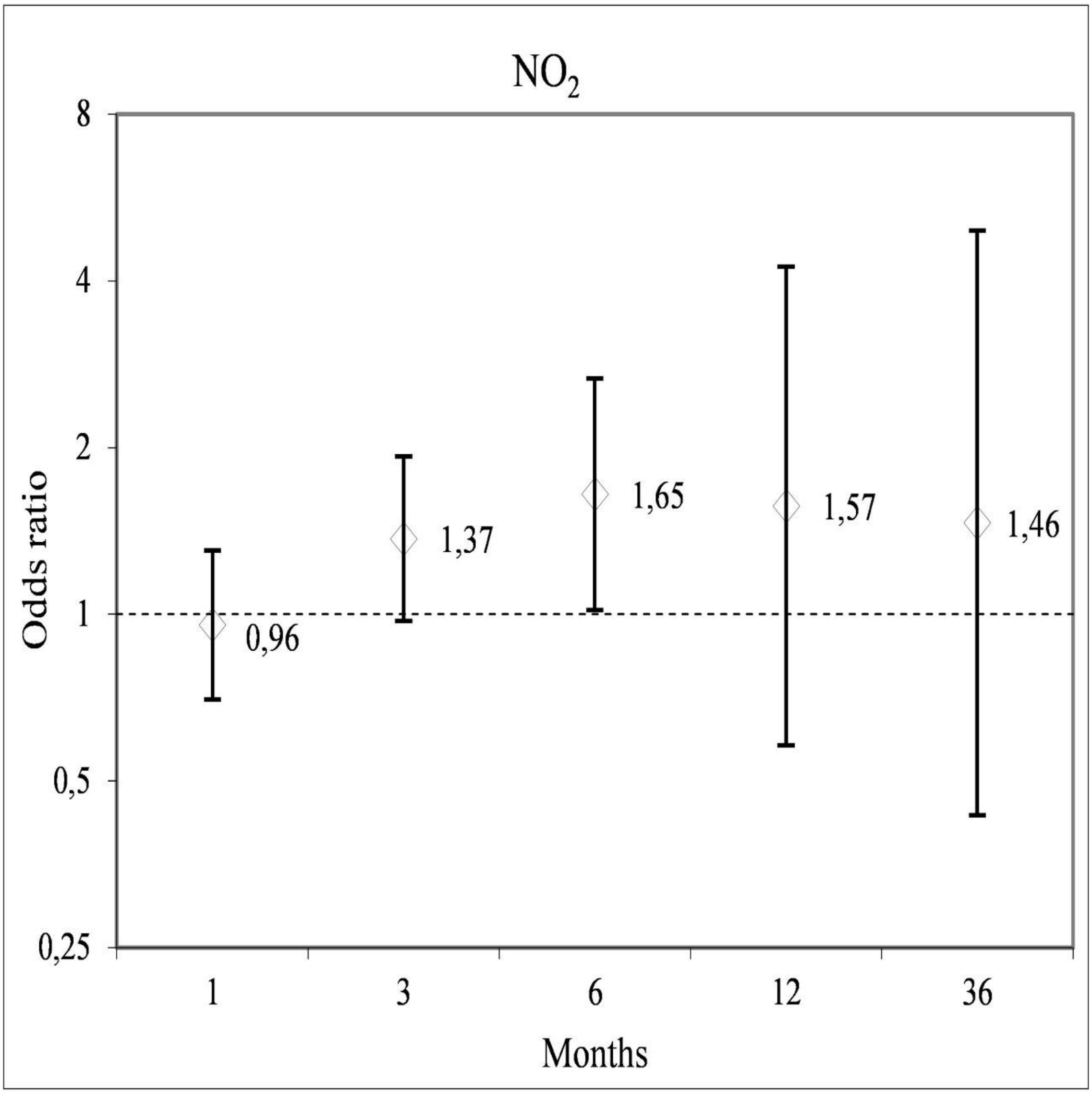

Supplementary figure 2. Effect of NO_2 on the development of chronic respiratory failure

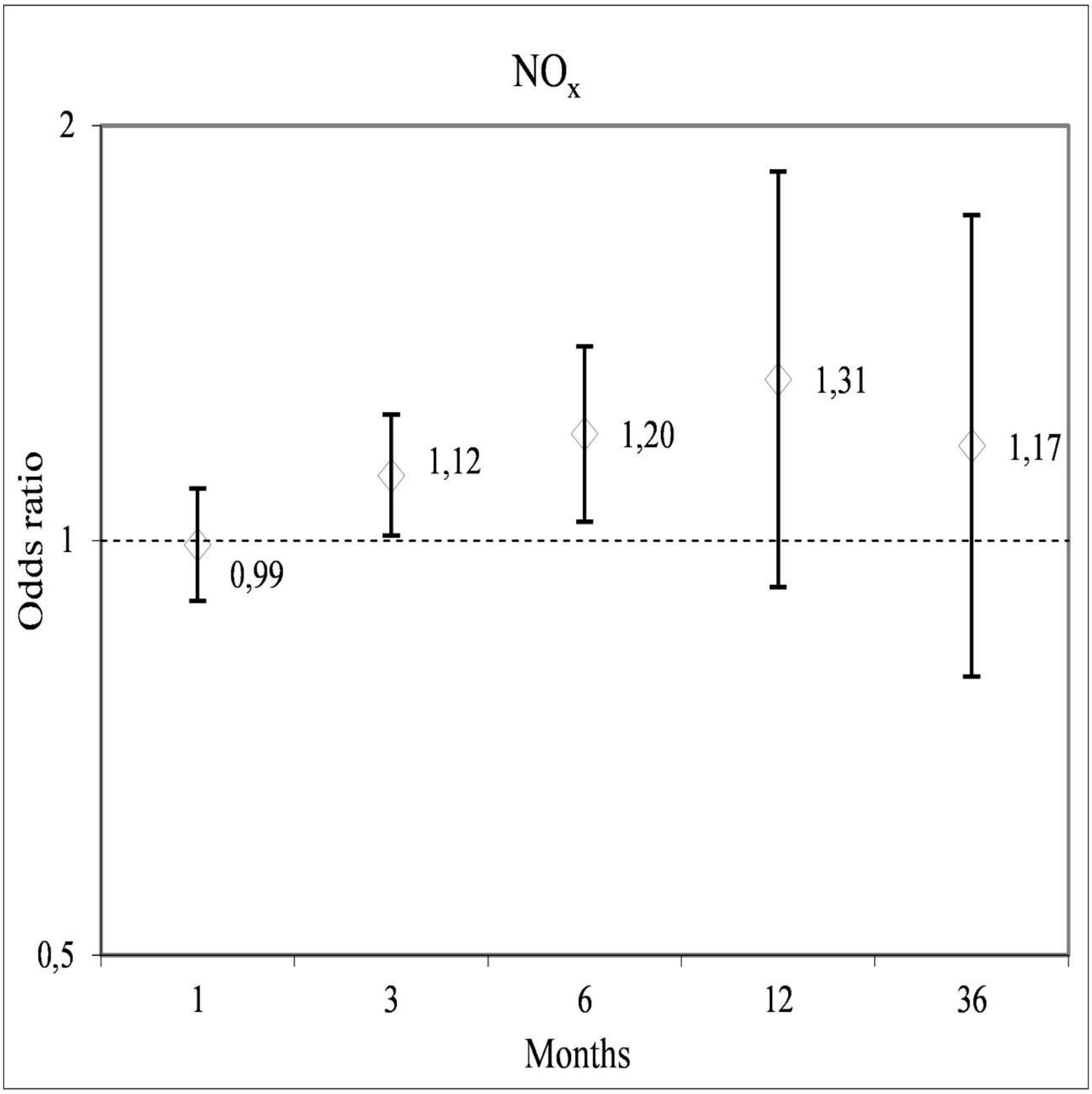

Supplementary figure 3. Effect of NO_x on the development of chronic respiratory failure

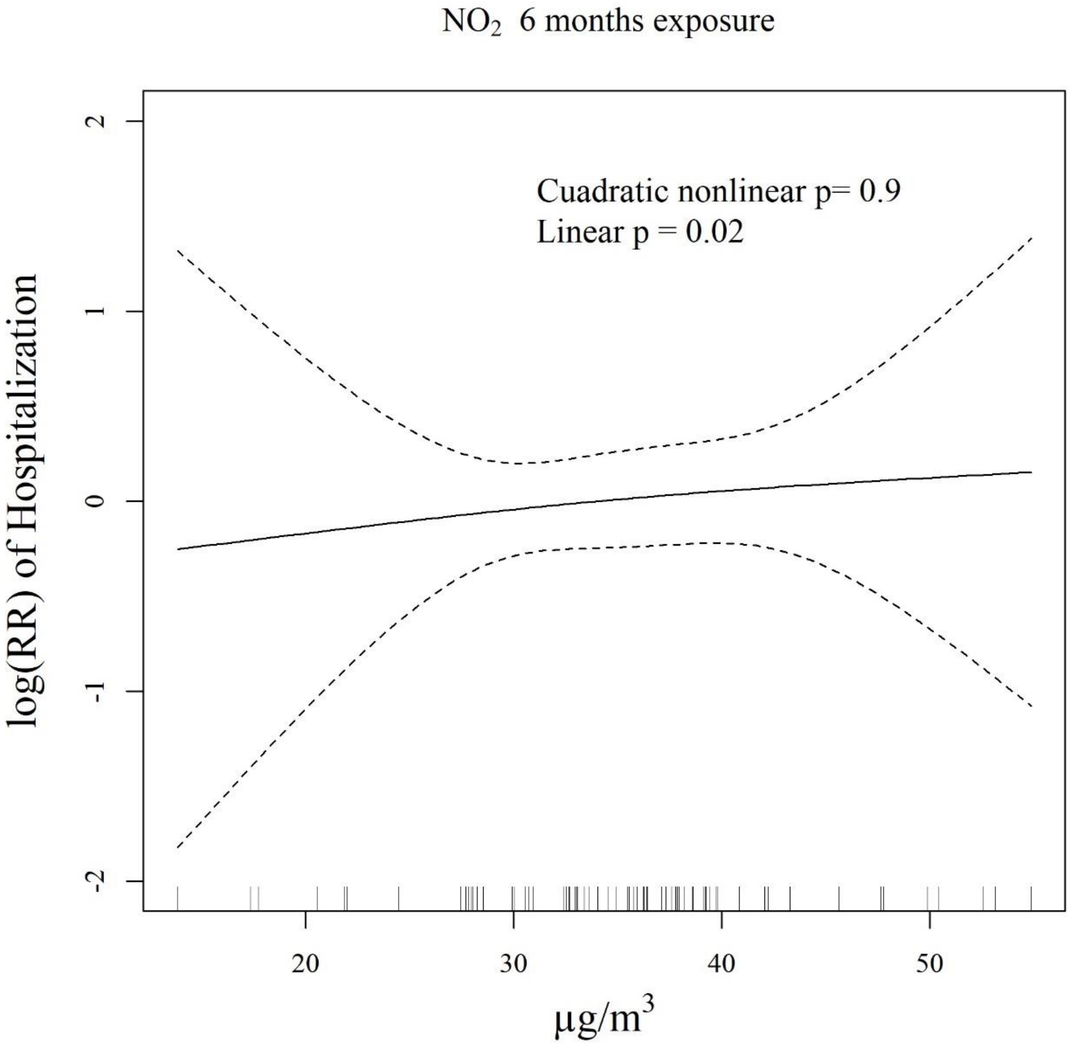

Supplementary figure 4. Effect of cumulative exposure to NO_2 during 6 months on the risk of hospitalization. Log (RR): logarithm of relative risk

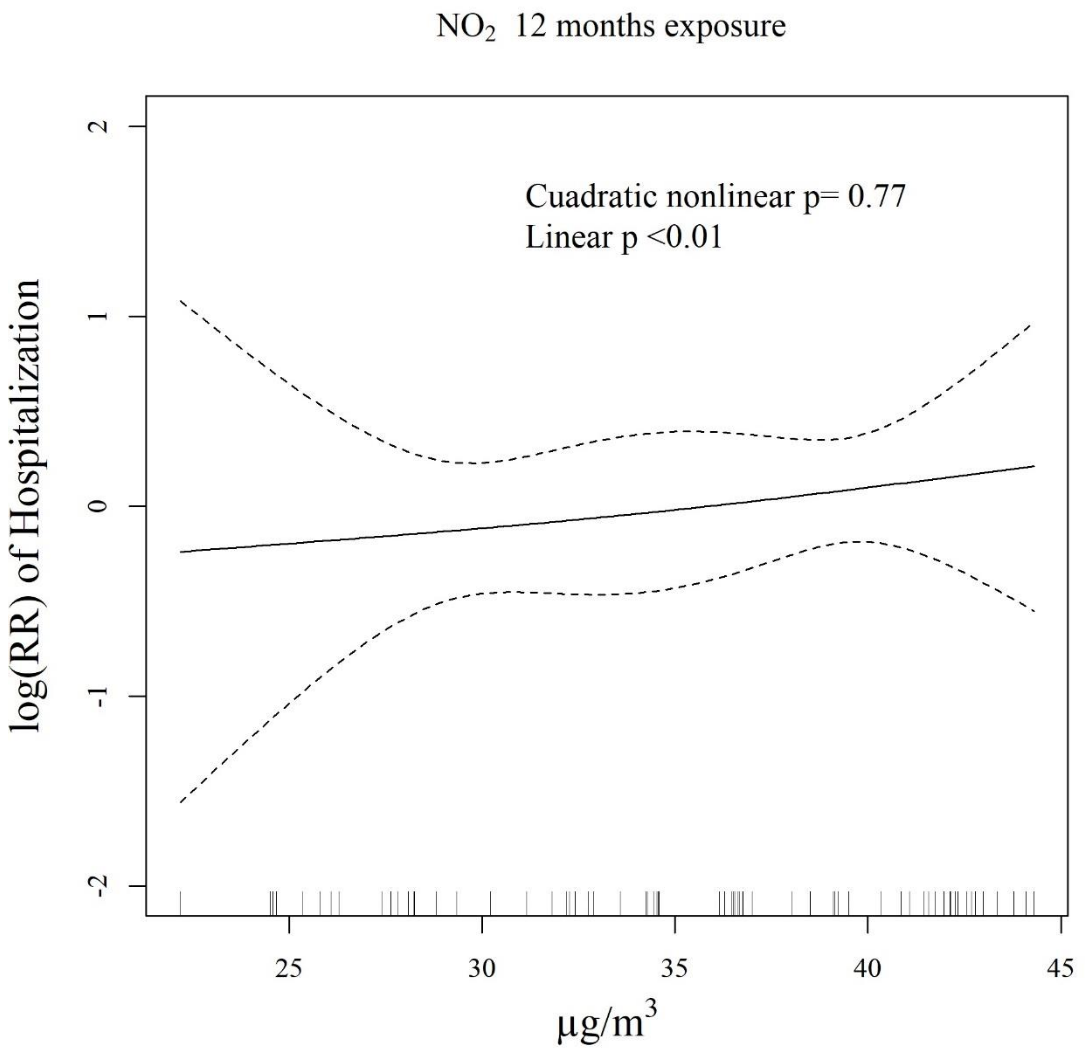

Supplementary figure 5. Effect of cumulative exposure to NO_2 during 12 months on the risk of hospitalization. Log (RR): logarithm of relative risk

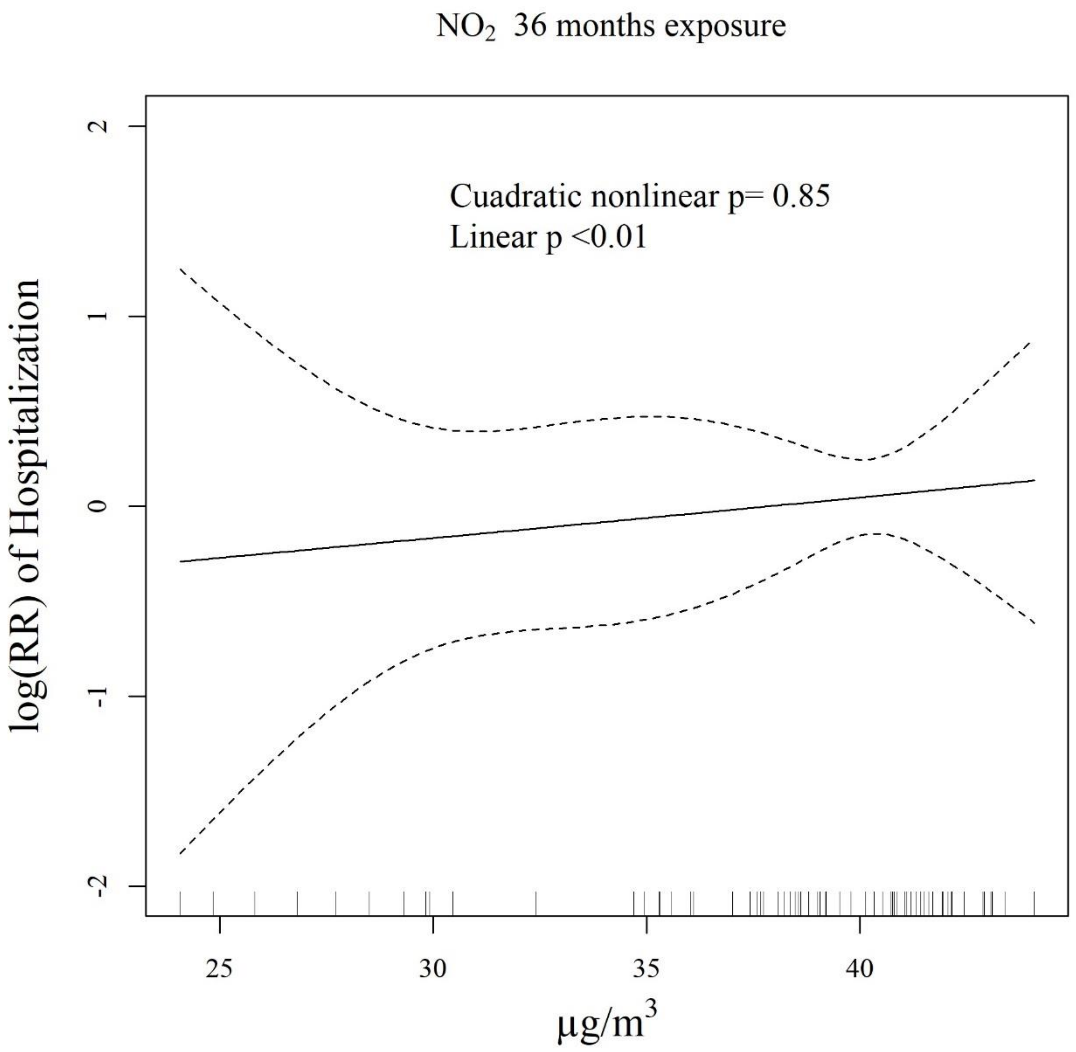

Supplementary figure 6. Effect of cumulative exposure to NO_2 during 36 months on the risk of hospitalization. Log (RR): logarithm of relative risk

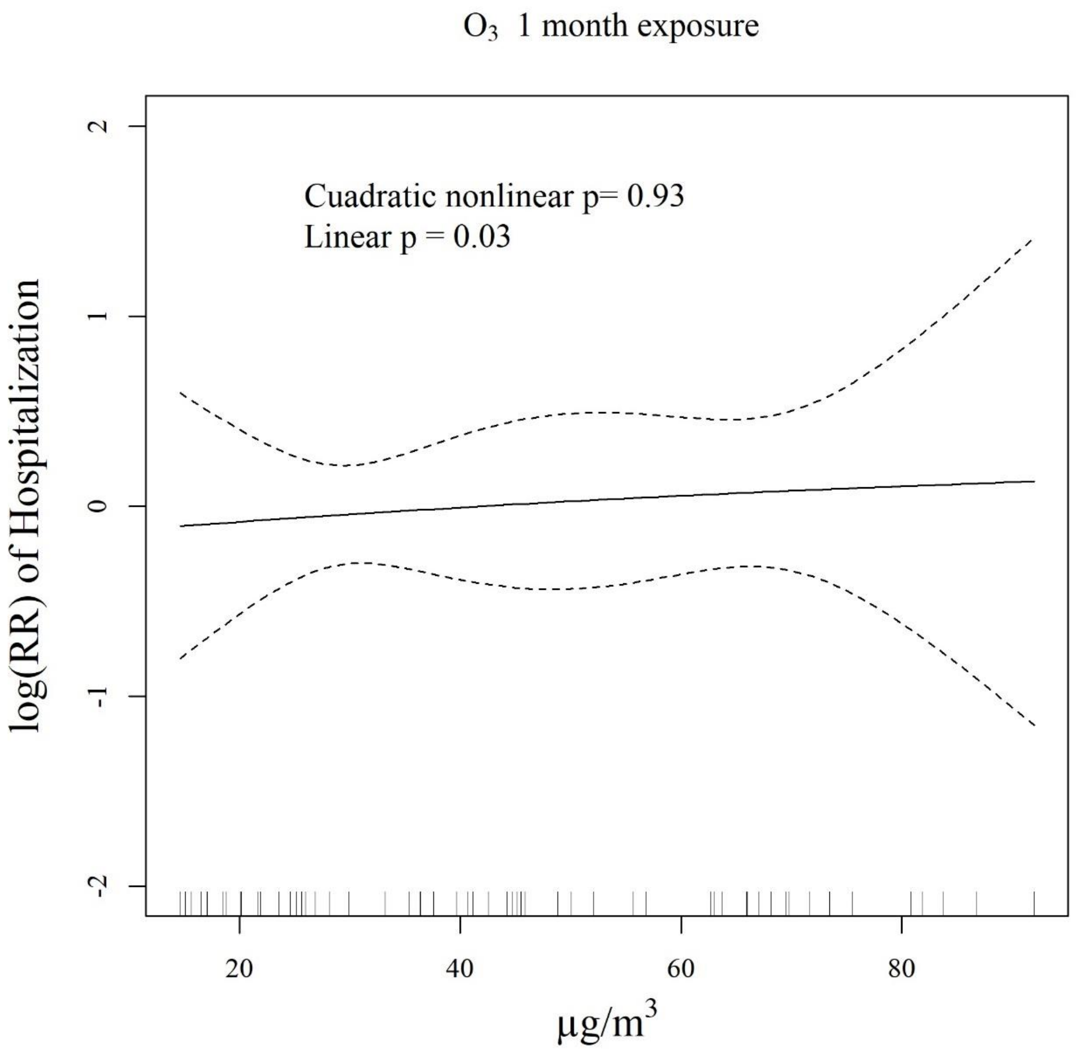

Supplementary figure 7. Effect of cumulative exposure to O_3 during one month on the risk of hospitalization. Log (RR): logarithm of relative risk

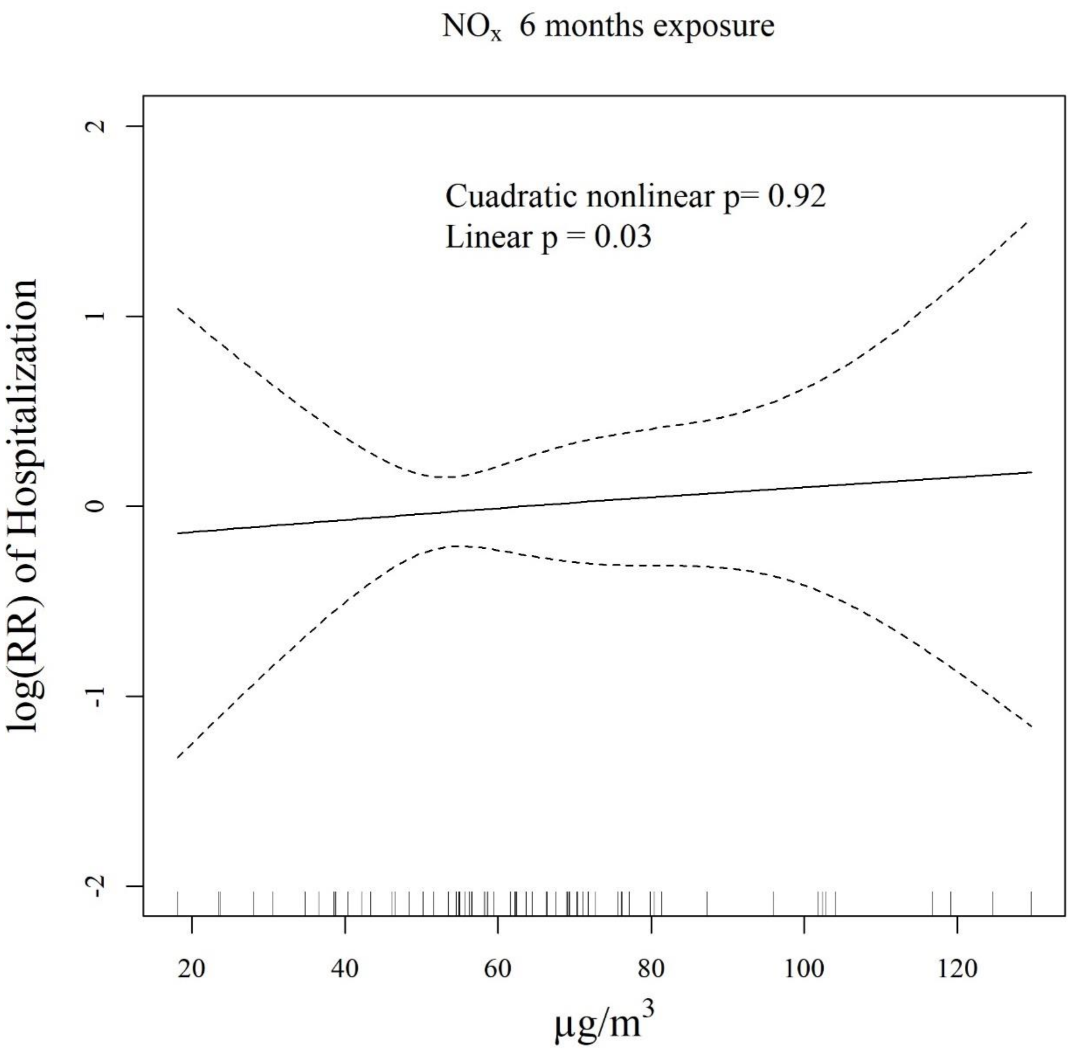

Supplementary figure 8. Effect of cumulative exposure to NO_x during 6 months on the risk of hospitalization. Log (RR): logarithm of relative risk

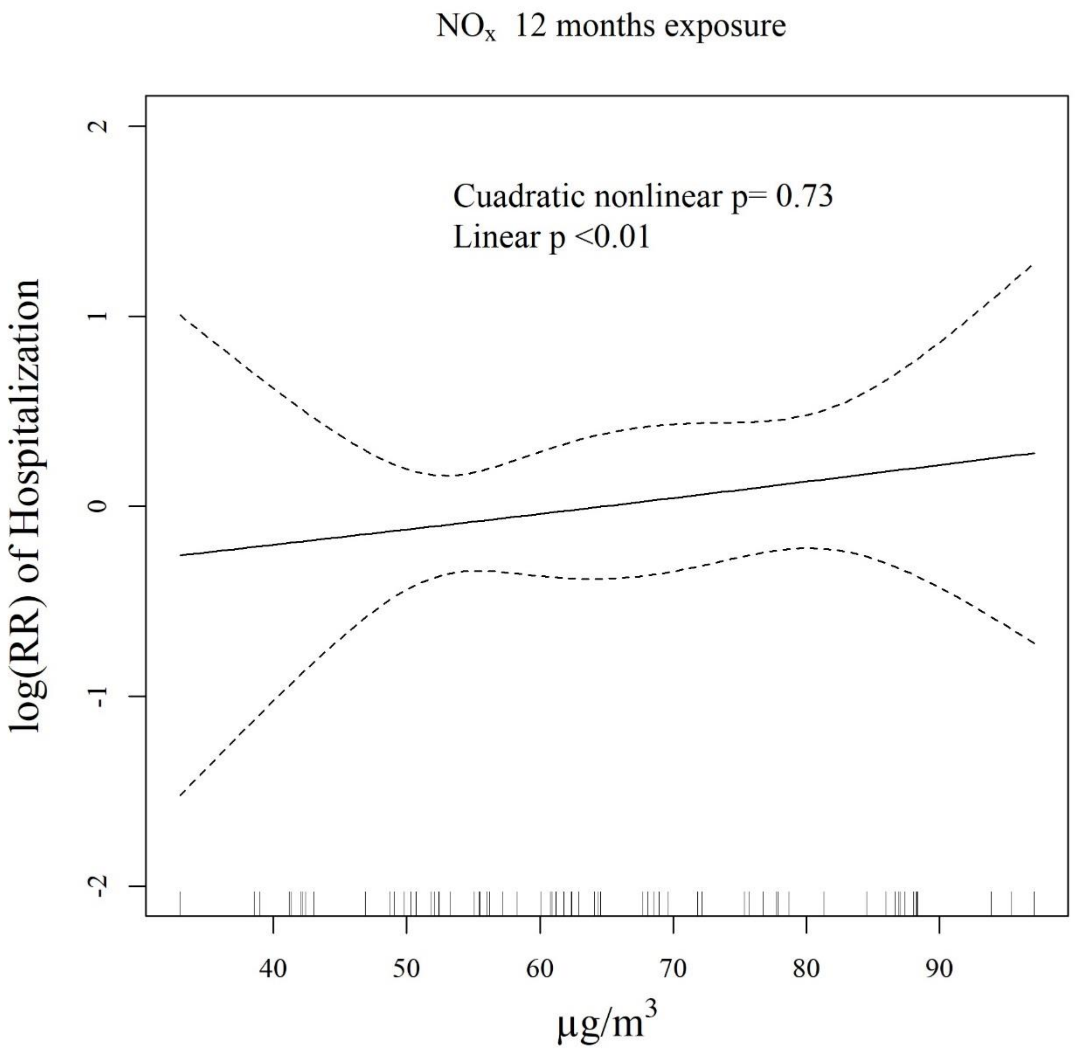

Supplementary figure 9. Effect of cumulative exposure to NO_x during 12 months on the risk of hospitalization. Log (RR): logarithm of relative risk

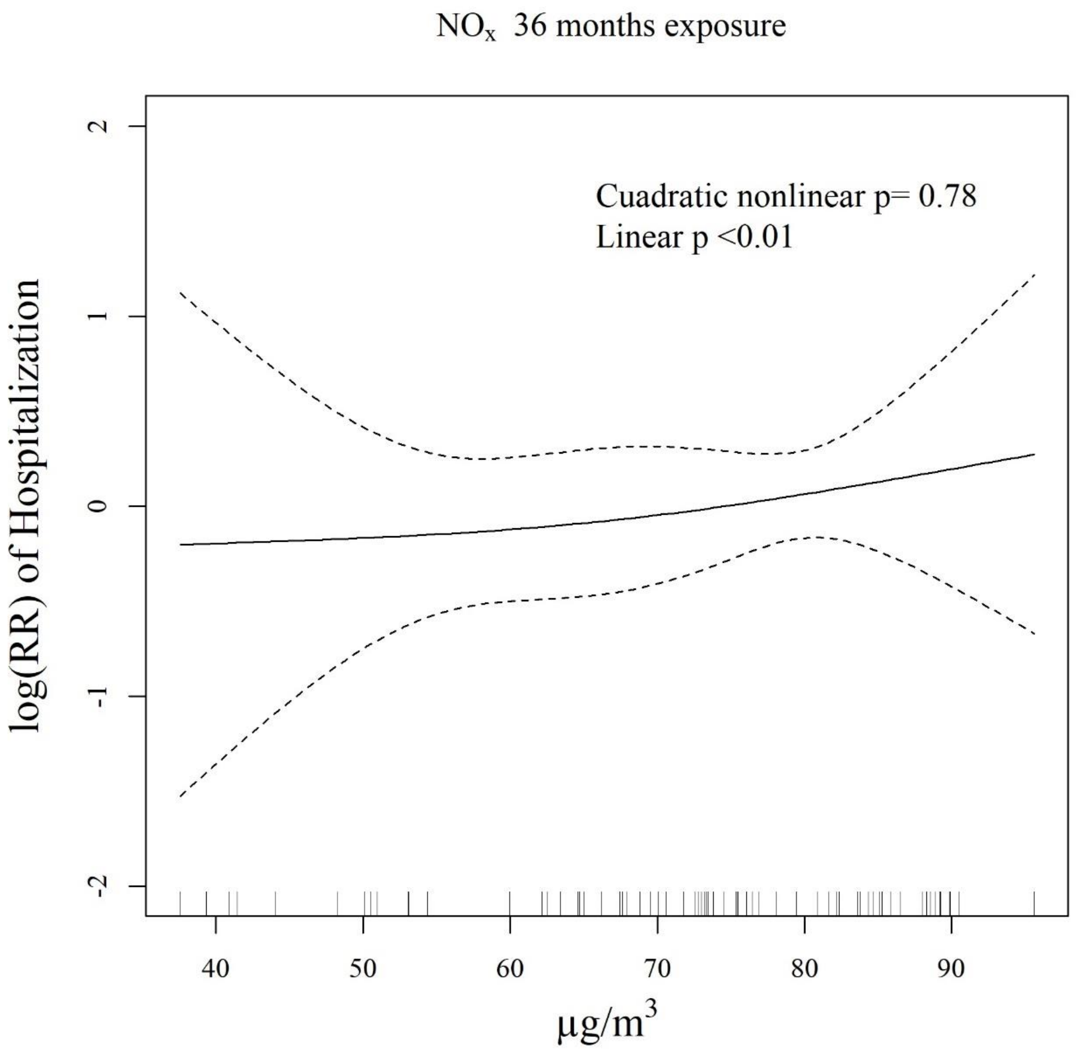

Supplementary figure 10. Effect of cumulative exposure to NO_x during 36 months on the risk of hospitalization. Log (RR): logarithm of relative risk

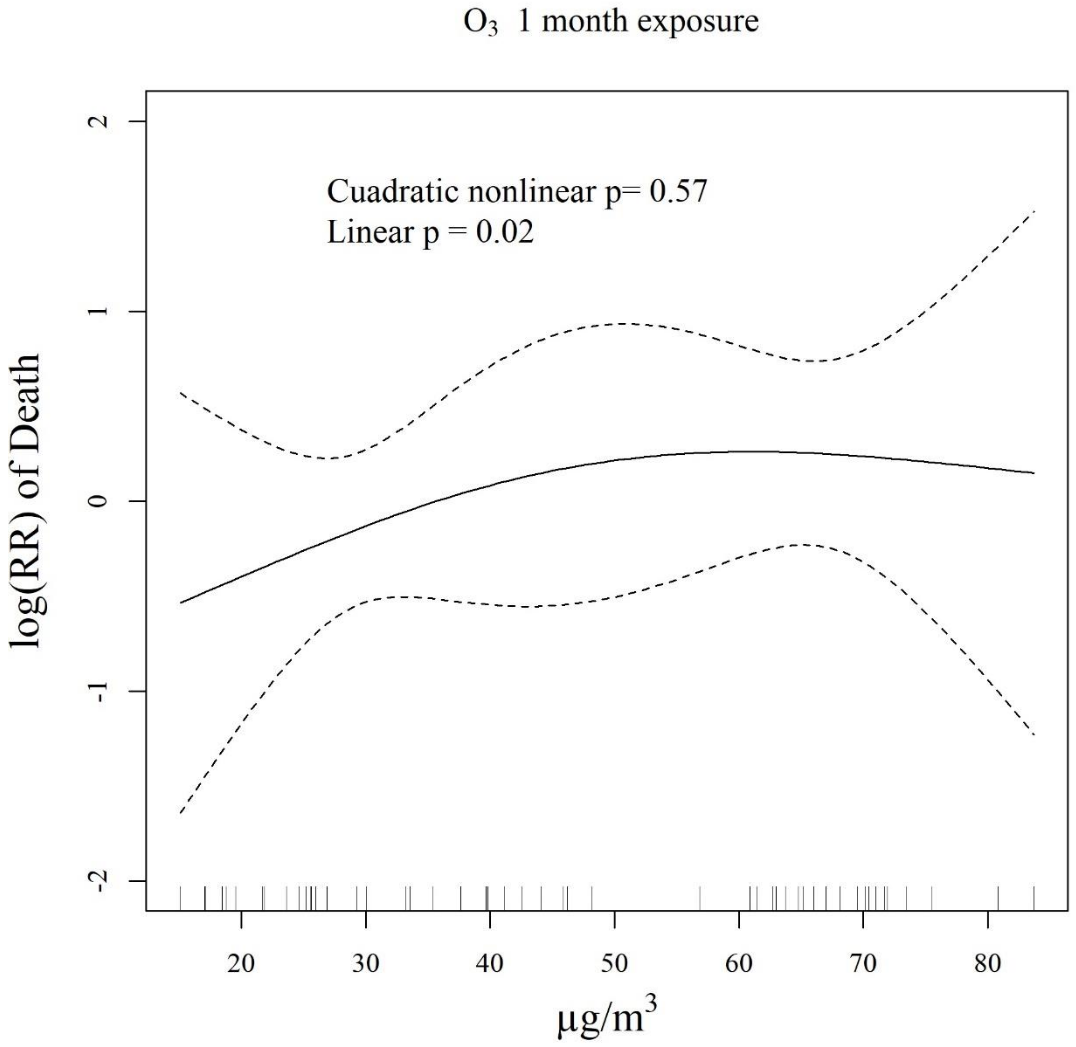

Supplementary figure 11. Effect of cumulative exposure to O_3 during one month on the risk of mortality. Log (RR): logarithm of relative risk

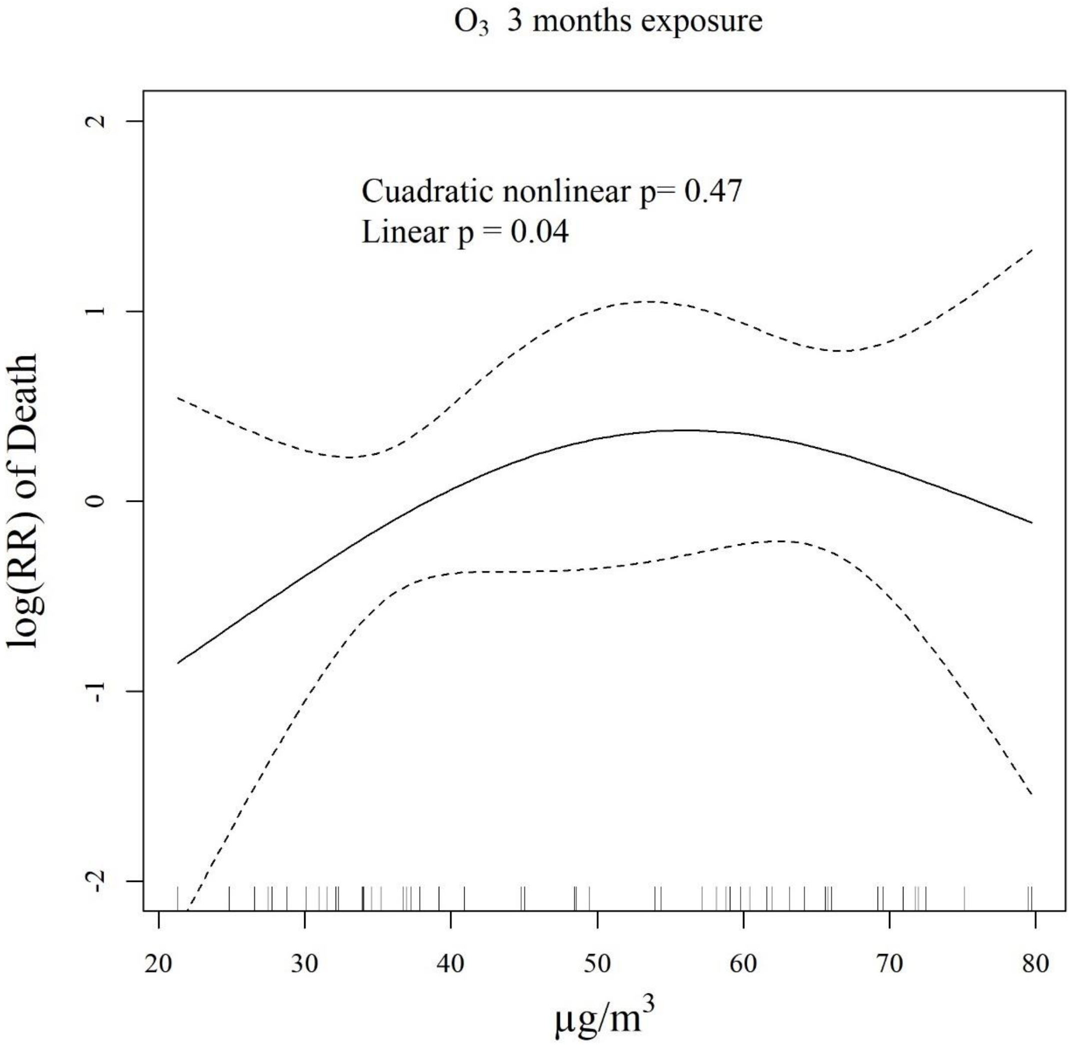

Supplementary figure 12. Effect of cumulative exposure to O_3 during three months on the risk of mortality. Log (RR): logarithm of relative risk

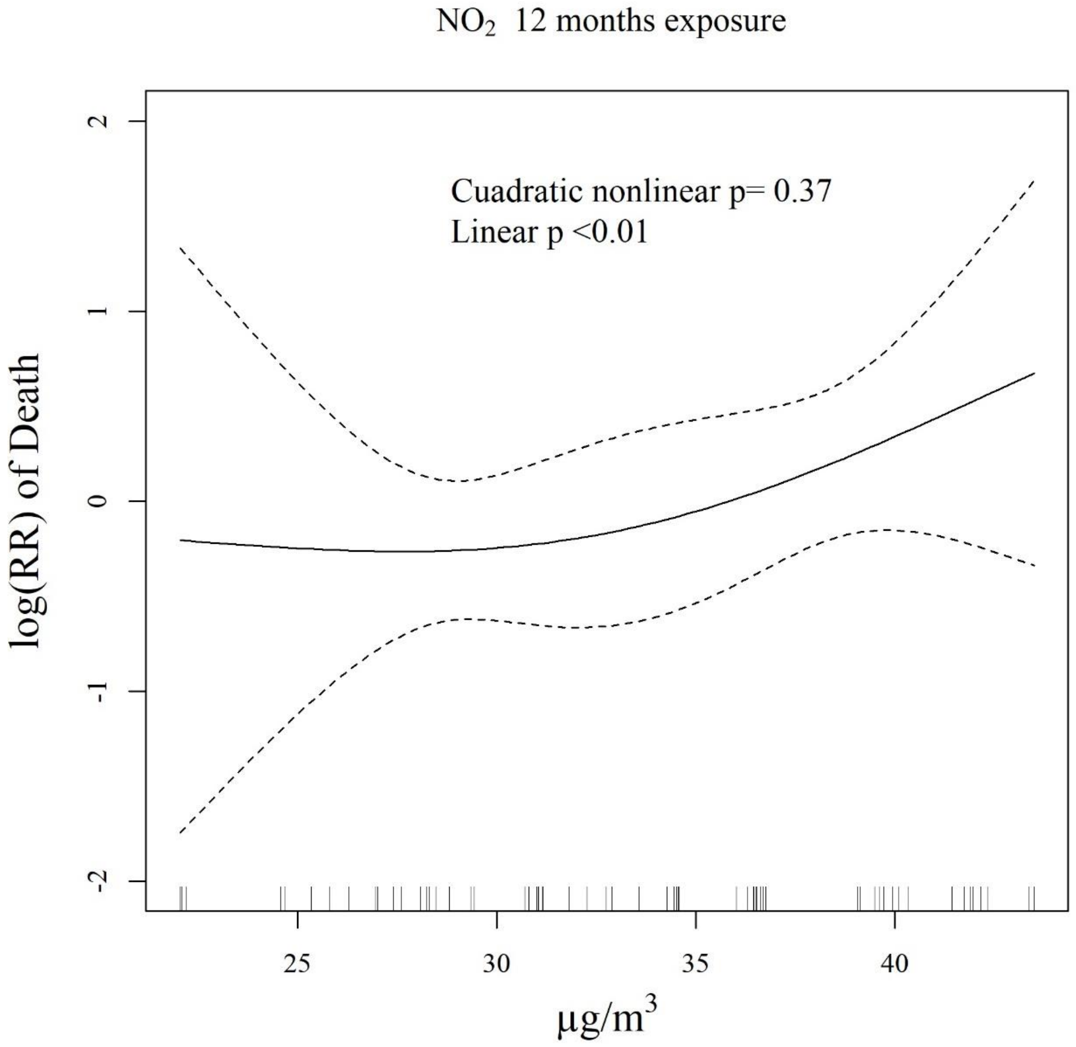

Supplementary figure 13. Effect of cumulative exposure to NO_2 during 12 months on the risk of mortality. Log (RR): logarithm of relative risk

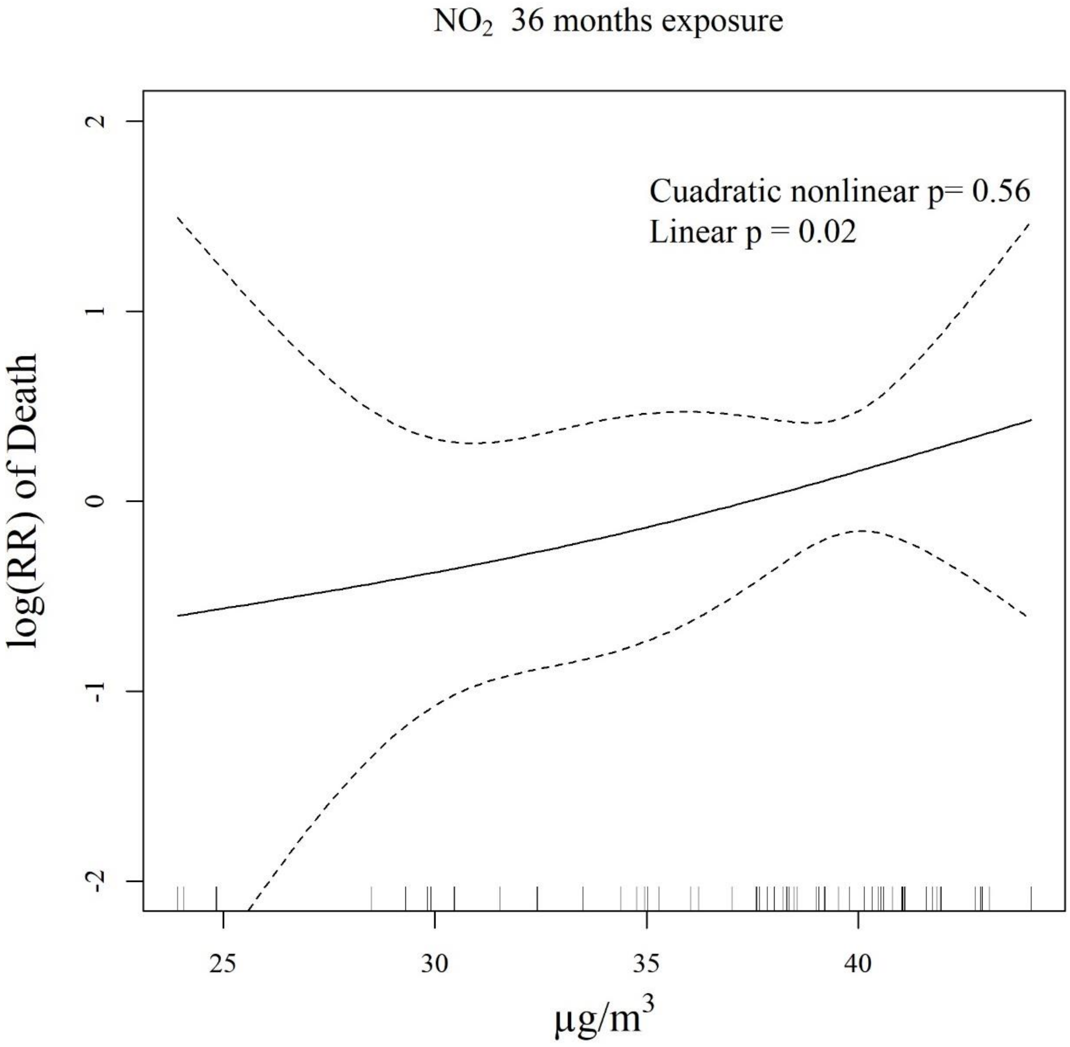

Supplementary figure 14. Effect of cumulative exposure to NO_2 during 36 months on the risk of mortality. Log (RR): logarithm of relative risk

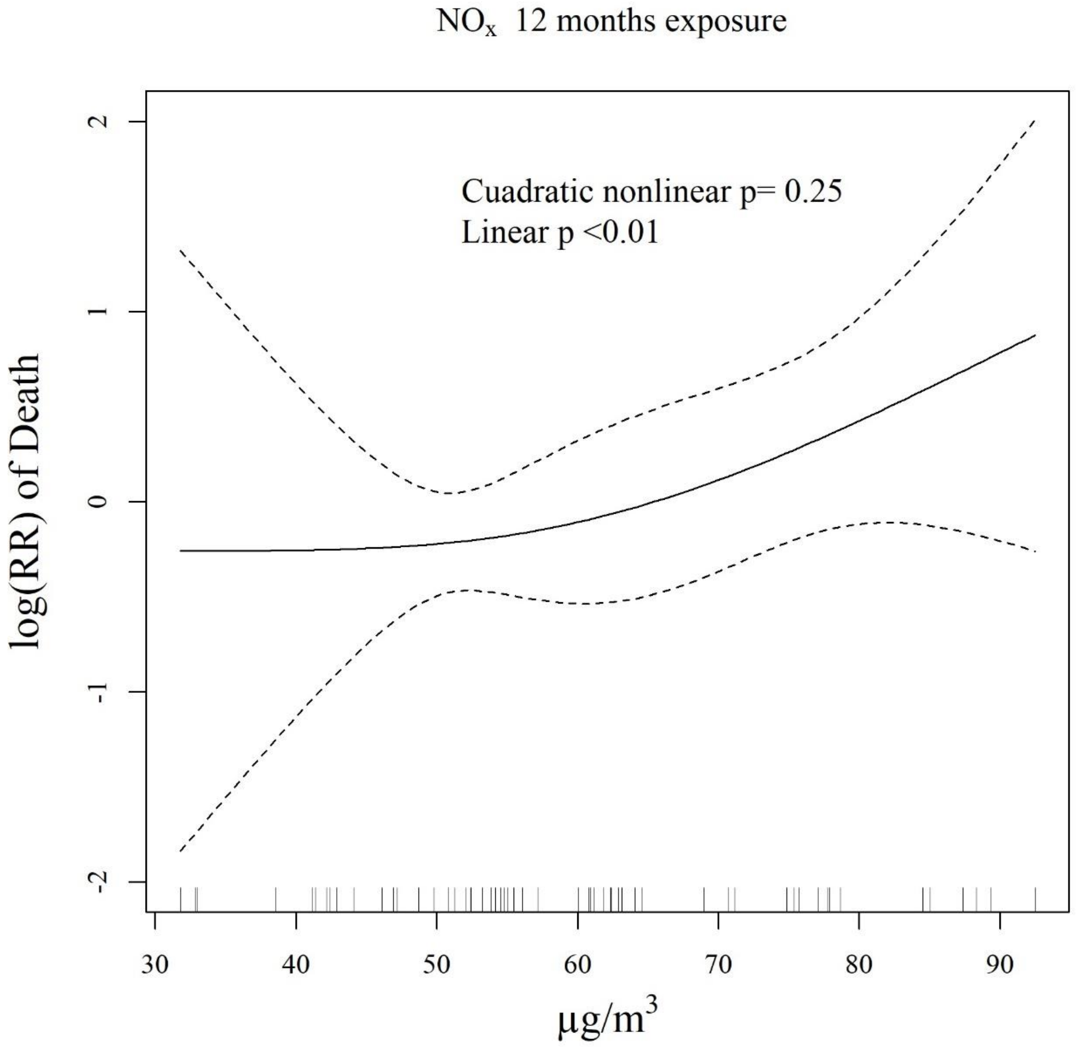

Supplementary figure 15. Effect of accumulative exposure to NO_x during 12 months on the risk of mortality. Log (RR): logarithm of relative risk

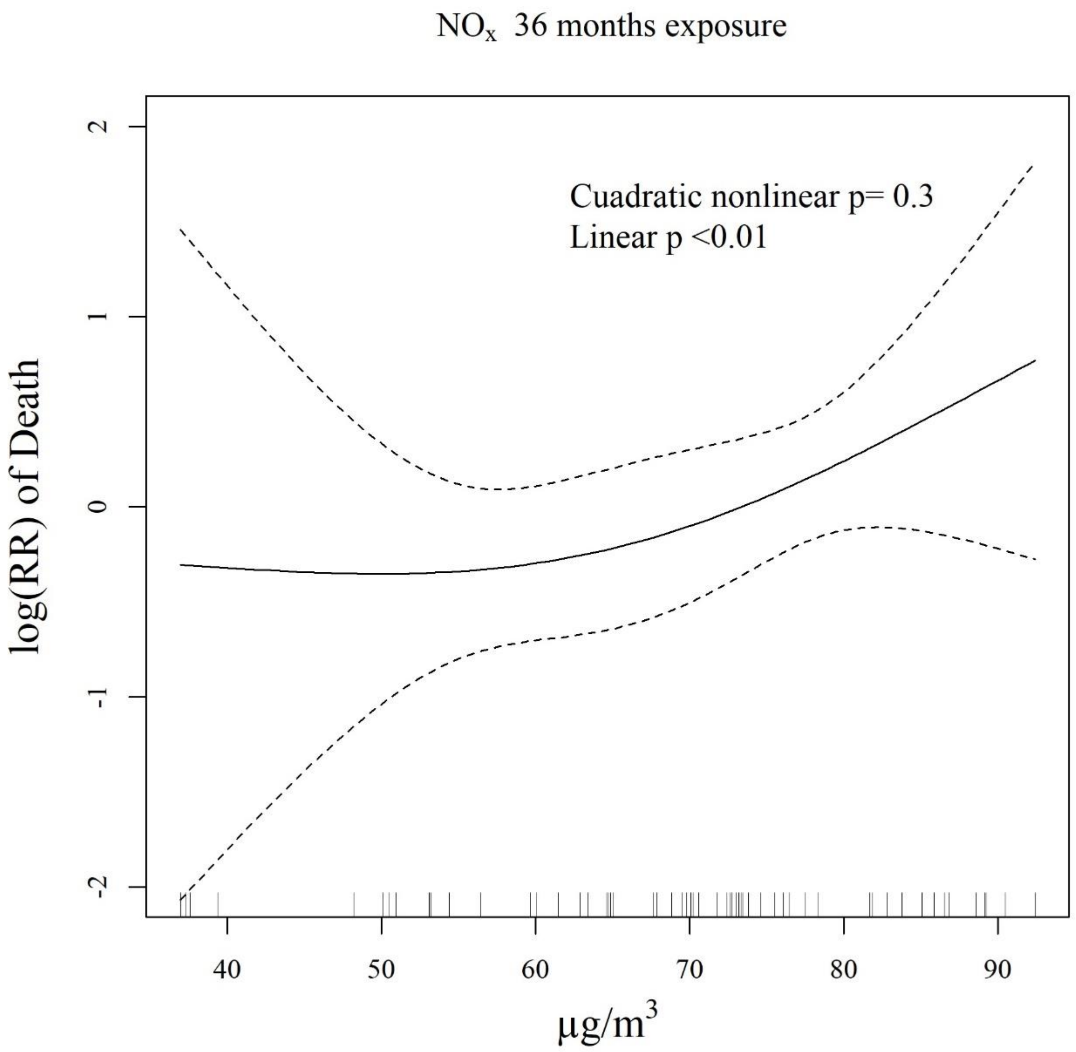

Supplementary figure 16. Effect of accumulative exposure to NO_x during 12 months on the risk of mortality. Log (RR): logarithm of relative risk
